# Premature Osteoblast Clustering by Enamel Matrix Proteins Induces Osteoblast Differentiation through Up-Regulation of Connexin 43 and N-Cadherin

**DOI:** 10.1371/journal.pone.0023375

**Published:** 2011-08-15

**Authors:** Richard J. Miron, Erik Hedbom, Sabrina Ruggiero, Dieter D. Bosshardt, Yufeng Zhang, Corinna Mauth, Anja C. Gemperli, Tateyuki Iizuka, Daniel Buser, Anton Sculean

**Affiliations:** 1 Department of Periodontology, School of Dental Medicine, University of Bern, Bern, Switzerland; 2 Department of Oral Surgery and Stomatology, School of Dental Medicine, University of Bern, Bern, Switzerland; 3 Department of Cranio-Maxillofacial Surgery, Bern University Hospital, Inselspital, Bern, Switzerland; 4 Institute Straumann AG, Basel, Switzerland; University of Southern California, United States of America

## Abstract

In recent years, enamel matrix derivative (EMD) has garnered much interest in the dental field for its apparent bioactivity that stimulates regeneration of periodontal tissues including periodontal ligament, cementum and alveolar bone. Despite its widespread use, the underlying cellular mechanisms remain unclear and an understanding of its biological interactions could identify new strategies for tissue engineering. Previous *in vitro* research has demonstrated that EMD promotes premature osteoblast clustering at early time points. The aim of the present study was to evaluate the influence of cell clustering on vital osteoblast cell-cell communication and adhesion molecules, connexin 43 (cx43) and N-cadherin (N-cad) as assessed by immunofluorescence imaging, real-time PCR and Western blot analysis. In addition, differentiation markers of osteoblasts were quantified using alkaline phosphatase, osteocalcin and von Kossa staining. EMD significantly increased the expression of connexin 43 and N-cadherin at early time points ranging from 2 to 5 days. Protein expression was localized to cell membranes when compared to control groups. Alkaline phosphatase activity was also significantly increased on EMD-coated samples at 3, 5 and 7 days post seeding. Interestingly, higher activity was localized to cell cluster regions. There was a 3 fold increase in osteocalcin and bone sialoprotein mRNA levels for osteoblasts cultured on EMD-coated culture dishes. Moreover, EMD significantly increased extracellular mineral deposition in cell clusters as assessed through von Kossa staining at 5, 7, 10 and 14 days post seeding. We conclude that EMD up-regulates the expression of vital osteoblast cell-cell communication and adhesion molecules, which enhances the differentiation and mineralization activity of osteoblasts. These findings provide further support for the clinical evidence that EMD increases the speed and quality of new bone formation *in vivo*.

## Introduction

Bone remodeling is a complex lifelong process that requires precise control of bone-resorbing osteoclasts and bone-forming osteoblasts for the maintenance of aging bone and repair of bone injuries. Osteoblasts are at the center of these processes by controlling matrix production and mineralization, receiving and processing mechanical and chemical signals to bone and most likely directing osteoclast function. This level of coordination demands sophisticated cell communication. Osteoblasts engage in a variety of cell-cell interactions including communication via soluble factors such as cytokines and growth factors as well as direct cell-cell adhesion molecules such as those forming adherens junctions and gap junctions [1], [2].

Adherens junctions are intercellular structures that are formed through hemophilic, calcium-dependent cell-cell adhesion via cadherins. These molecules constitue a class of 30 single chain integral membrane glycoproteins composed of a long N-terminal extracellular domain, a single transmembrane domain, and a small intracellular C-terminal tail [3]. The intracellular domain anchors to the actin cytoskeleton via multiple protein complexes including α- and β-catenin which control Wnt signaling [4]. Two cadherins are predominantly expressed in osteoblasts, N-cadherin (N-cad) and cadherin-11. Knockout models have indicated that the loss of N-cad disrupts cell-cell adhesion more severely than the loss of cadherin-11 in osteoblasts [5]. Cadherins also play essential roles in fetal development of mesenchymal tissues including morphogenesis, osteogenesis and chondrocyte condensation [3], [6], [7].

Gap junctions are aqueous transmembrane channels that connect the cytoplasm of two adjacent cells and allow the diffusion of small molecules with a molecular mass of less than 1 kDa such as small metabolites, ions, and intracellular signaling molecules (calcium, cAMP, and inositol triphosphate) to pass through [8]. Each gap junction pore is formed of connexins which oligomerize to form hemichannels that tightly dock with hemichannels from the adjacent cell [9], [10]. The connexin superfamily includes more than 20 genes, whose products have different molecular properties that influence the permeability of each gap junction [9]. The main connexin in osteoblasts, connexin 43 (cx43) is the most abundant gap junction protein in the skeleton [11]. The role of cx43 in osteoblast differentiation and mineralization has been demonstrated by *in vitro* experiments. The inhibition of cx43 gene expression by antisense transfection or knockout-cx43 has consistently been associated with loss of gap junctional coupling and reduced osteoblast differentiation potential as assessed by downregulation of alkaline phosphatase, osteocalcin, bone sialoprotein and mineralization [12], [13], [14], [15]. In contrast, overexpression of cx43 results in an enhancement of gap junctional intercellular communication and expression of an osteogenic phenotype [12], [16], [17].

The clearest demonstration of the essential role of cx43 in bone formation has been observed in knockout mice lacking cx43 [15]. These animals exhibit profound defects in intramembranous and endochondral ossification of the skeleton, leading to skull abnormalities, brittle, misshapen ribs and delayed mineralization [15], [18]. They display many similarities with the phenotypes reported from human oculodentodigital dysplasia (ODDD), an autosomal dominant disease caused by any one of over 60 mutations in the gene GJA1 encoding Cx43 [19], [20], [21]. Recent research also suggests a role in wound healing [22]. An improved understanding of the regulation of gap junctions in bone could also provide further insights into regulatory mechanisms of osteoblast differentiation for further therapy in the treatment of bone loss diseases such as osteoporosis and periodontitis [23], [24], [25], [26].

One procedure for the regeneration of bone is the application of an enamel matrix derivative (EMD) which has been used clinically for the treatment of various types of bony defects located at periodontitis diseased teeth [27], [28]. EMD is extracted from developing porcine teeth, the major component of which are amelogenins, a family of hydrophobic proteins that account for more than 90% of the total protein content [29]. The remaining components of EMD include enamelins, such as proline-rich enamelin, sheathlin, tuftelin, amelotin and apin [30]. The direct effects of EMD on bone regeneration have primarily been evaluated in periodontal intrabony and class II furcation defects [31], [32], [33], [34]. However, findings from *in vitro* and *in vivo* experiments indicate that EMD may also influence healing/regeneration of non-tooth related bone defects. *In vitro* studies with human, rat and mouse osteoblasts showed increased proliferation and/or differentiation in the presence of EMD [30], [35], [36], [37], [38]. *In vivo* treatment of perforated rat femurs with EMD significantly increased newly formed bone in 7 days when compared to untreated perforated femurs [39].

Despite the widespread use of EMD, the underlying cellular mechanisms remain unclear and an understanding of its biological interactions could identify new strategies for tissue engineering. Previous *in vitro* research showed that EMD promoted osteoblast clustering at early time points [40]. The aim of the present study was to evaluate the influence of cell clustering on vital osteoblast cell-cell communication and adhesion molecules, cx43 and N-cad.

## Materials and Methods

### Surface Coating with EMD

EMD was prepared according to Institut Straumann AG standard operating protocols. 30 mg of EMD was dissolved in 3 ml of 4°C sterile 0.1% acetic acid. For experiments, stock EMD was diluted 100× in 0.1 M carbonate buffer at 4°C giving a working solution of 100 µg/ml. 1 ml of EMD solution was poured onto each 24 well culture dishes and incubated overnight at 4°C. Following incubation, dishes were rinsed with 1 ml phosphate buffered saline (PBS) twice at 4°C. Uncoated 24 well culture dishes were used as a control.

### Osteoblast Isolation and Differentiation

Human bone chips were cultured from an explants model [41] under sterile conditions at the University of Bern Dental Clinic under a protocol approved by the Ethics Committee, University of Bern. Primary human osteoblasts were removed from the tissue culture plastic using trypsin solution (Invitrogen, Basel, Switzerland). Osteoblasts used for experimental seeding were from passages 4–6. During cell seeding, α-MEM medium was supplemented with 50 µg/ml ascorbic acid and 2 mM β-glycerophosphate to promote osteoblast differentiation. Primary osteoblasts were seeded at a density of 10,000 cells in 24 well culture plates for all experiments. For experiments lasting longer than 5 days, medium was replaced twice weekly.

### Immunofluorescence

Osteogenic cells were plated at a density of 10,000 cells in 24 well culture plates. At multiple time points ranging from 1 to 14 days cells were fixed in 4% buffered formalin, followed by three 5 min washes in PBS. Cells were permealized with Triton X-100 for 5 minutes, followed by staining with antibodies against connexin 43 (sc-9059) or N-cadherin (sc-7939) (Santa Cruz Biotechnologies Inc., Heidelberg, Germany) antibodies, followed by a goat anti-rabbit IgG conjugated to texas red at a dilution of 1∶200 in 0.5% PBS/bovine serum albumin (Invitrogen). The dilution of each antibody was titrated to determine the optimal concentration. Prior to viewing, samples were mounted with Vectashield containing DAPI nuclear staining (Vector). Images were captured using an Olympus BX-51 (Center Valley, Pennsylvania) microscope with a ProgRes CT3 digital camera (Jenoptik Laser, Optik, Systeme GmbH, Jena, Germany).

### Real Time RT-PCR

Total RNA was isolated using TRIZOL reagent and RNAeasy Mini kit (QIAGEN, Basel, Switzerland) at time points 1, 2, 3, 5, 7, 10 and 14 days. Primer and probe sequences for genes encoding cx43 (Hs00748445_s1), N-cad (Hs00983056_m1), Runx2 (Hs00231692_m1), collagen1α1 (COL1A1, Hs01028970_m1), osteocalcin (OC, Hs01587814_g1), bone sialoprotein (BSP, Hs00173720_m1) and GAPDH (Hs03929097_g1) were purchased as pre-designed gene expression assays (Applied Biosystems, Rotkreus, Switzerland). Real-time RT-PCR was performed using 20 µl final reaction volume of TaqMan®'s One step Master Mix kit (Applied Biosystems). RNA quantification was performed using a Nanodrop 2000c (Thermo Scientific, Waltham, MA, USA) and 100 ng of total RNA was used per sample well. The ΔΔCt method was used to calculate gene expression levels normalized to GAPDH values.

### Western Blot Analysis

Samples extracted using RIPA buffer were separated by SDS-PAGE and blotted to nitrocellulose membrane as previously described (Brellier et al. 2011). After a blocking step in 1% milk, membranes were incubated with rabbit polyclonal antibodies for cx43 and N-cad. They were then incubated for 1 h with anti-rabbit IgG coupled to horseradish peroxidase. Blots were developed using ECL reagent (GE Healthcare, Otelfingen, Switzerland) and exposed to Fuji Medical X-ray Film (Fujifilm Europe GmbH, Dusseldorf, Germany).

### Alkaline Phosphatase activity

Alkaline Phosphatase activity was monitored using fast violet B salt kit (procedure No. 85, Sigma Aldrich). Briefly, 1 fast violet B salt capsule was dissolved in 48 ml of distilled water and 2 ml of naphtol AS-MX phosphate alkaline solution. Osteoblasts were fixed by immersing in a citrate-buffered acetone solution (2 parts citrate, 3 parts acetone) for 30 s and rinsed in deionized water for 45 s. Samples were then placed in alkaline phosphatase stain for 30 min protected from light. Following 2 min of rinsing in deionized water, slides were treated with Mayer's hematoxylin solution for 10 min. Light microscopic recording was performed using a ProgRes® C5 digital camera connected to a Zeiss Axioplan microscope (Carl Zeiss, Göttingen, Germany). All images were captured using pre-determined light intensity at the same magnification. Image pro plus thresholding software was used to generate percent stained values for each field of view.

### Osteocalcin Staining

To determine extracellular osteocalcin deposition, osteoblasts were seeded at a density of 10,000 cells in 24 well culture plates. At time points ranging from 5 to 14 days cells were fixed in 4% buffered formalin, followed by three 5 min washes in PBS. Cells were stained with osteocalcin (sc-7449, Santa Cruz Biotechnology, Santa Cruz, CA) antibody, followed by a goat anti-rabbit IgG conjugated to texas red at a dilution of 1∶200 in 0.5% PBS/Bovine Serum Albumin (Invitrogen). Images were captured using an Olympus BX-51 (Center Valley, Pennsylvania) microscope with a ProgRes CT3 digital camera.

### Von Kossa Staining

Von Kossa staining was performed to determine the presence of mineralization. Osteoblasts were seeded at a density of 10,000 cells per 24-well plate pre-coated with and without EMD. At time points 5, 10 and 14 days, cells were fixed in 4% PFA for 15 min and stained with 2% aqueous silver nitrate for 30 minutes under bright sunlight. Light microscopic recording was performed using a ProgRes® C5 digital camera connected to a Zeiss Axioplan microscope (Carl Zeiss). All images were captured using pre-determined light intensity at the same magnification. Image pro plus thresholding software was used to generate percent stained values for each field of view. The size of bone nodules was measured as previously described [40].

### Statistical analysis

All samples from immunofluorescence, real-time PCR, alkaline phosphatase staining and von Kossa staining were assayed in triplicate with 3 independent experiments performed. Data were displayed by mean +/− SE and analyzed for statistical significance using 2-way ANOVA with Bonferri test (p<0.05) using Graphpad Software v. 4 (Graphpad Software, La Jolla, CA, USA).

## Results

### Cell clustering, cx43 and N-cad expression

**Figure 1:**
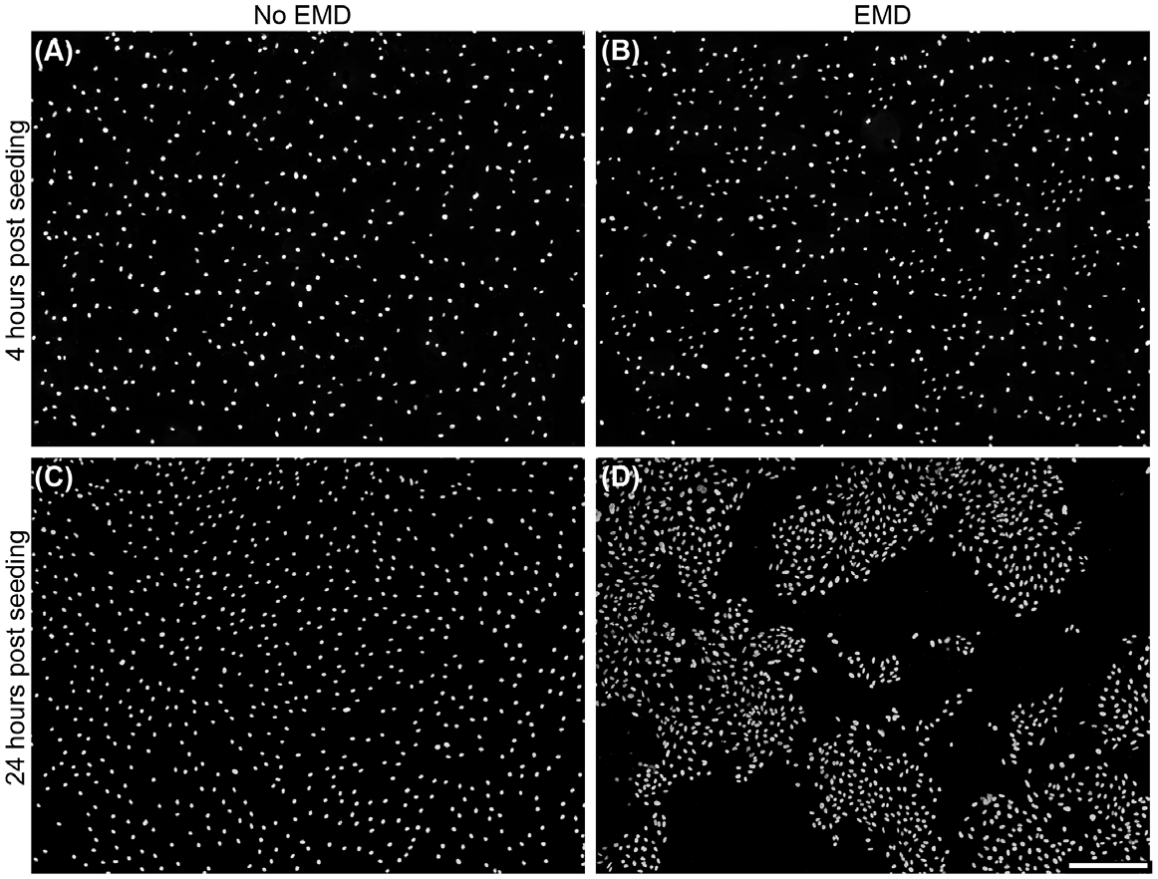
EMD promotes clustering of primary human osteoblasts on EMD-coated samples. On control and EMD coated samples, cells were evenly distributed as shown with DAPI staining at 4 hours post seeding (A–B). However after 24 hours, clustering of cells was apparent after 24 hours on EMD coated samples (D) when compared to control samples (C). (bar = 500 µm).

In order to visualize early formation of cell clusters, nuclear staining was employed. Primary osteoblasts attached well on both surfaces at 4 hours (Fig. 1A–B), however cells seeded on samples pre-coated with EMD began to form cell clusters after 24 hours (Fig. 1D) when compared to control (Fig. 1C).

**Figure 2:**
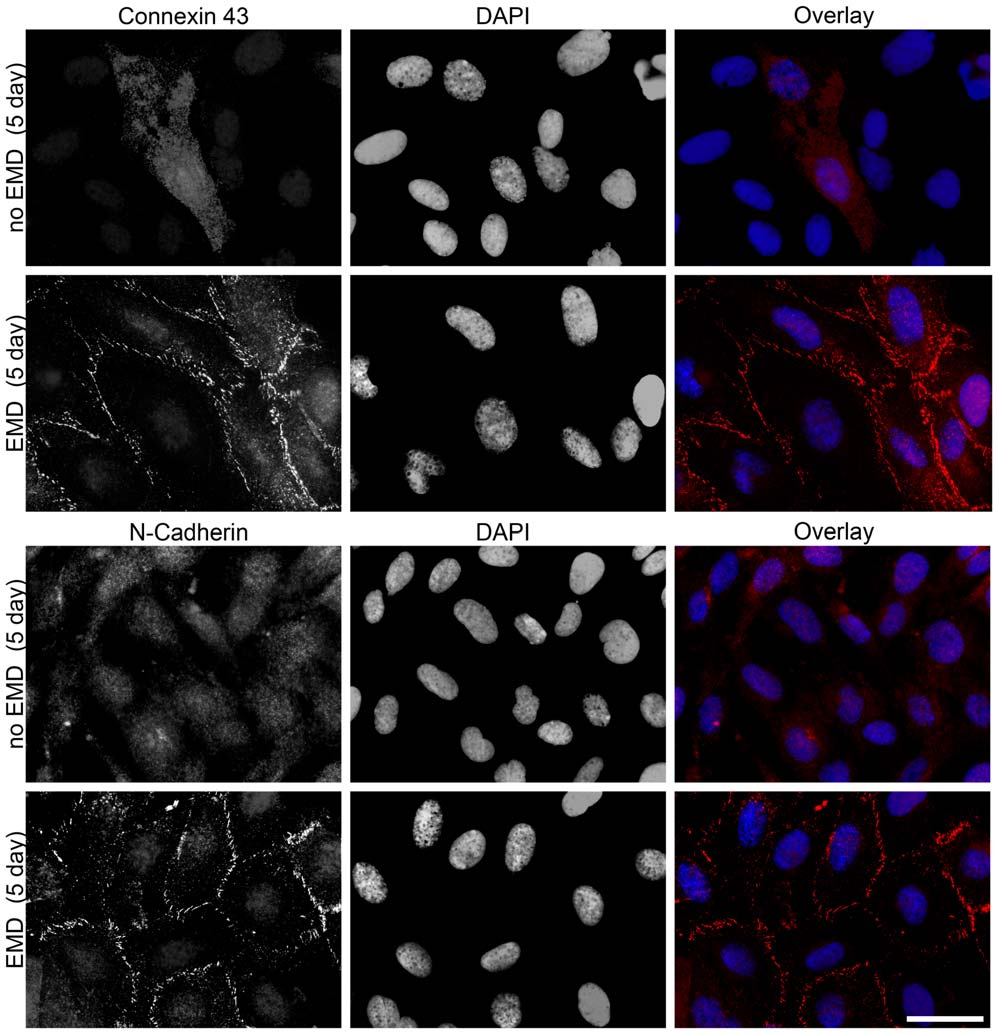
EMD promotes expression of connexin 43 and N-cadherin in cell clusters. At time point 5 days post seeding, primary human osteoblasts were stained for connexin 43 or N-cadherin (red), and nuclei (blue). Expression of connexin 43 and N-cadherin significantly increased on cell membranes of EMD coated samples. (bar = 50 µm).

Following cell clustering on EMD-coated samples, cells were immunolabeled for expression of cx43 and N-cad. Cells seeded on EMD-coated samples demonstrated elevated levels of cx43 and N-cad expression when compared to control samples (Fig. 2). At early time points (2–3 days), expression of cx43 and N-cad was found throughout the cytoplasm as well as on the cell-membranes on EMD-coated samples (not shown). Low levels were observed in control samples. After 5 days, virtually all cells expressed high levels of cx43 and N-cad on their cell membranes in EMD-coated samples. Elevated expression of cx43 was maintained up to 14 days post seeding at which point control samples also demonstrated high levels.

**Figure 3:**
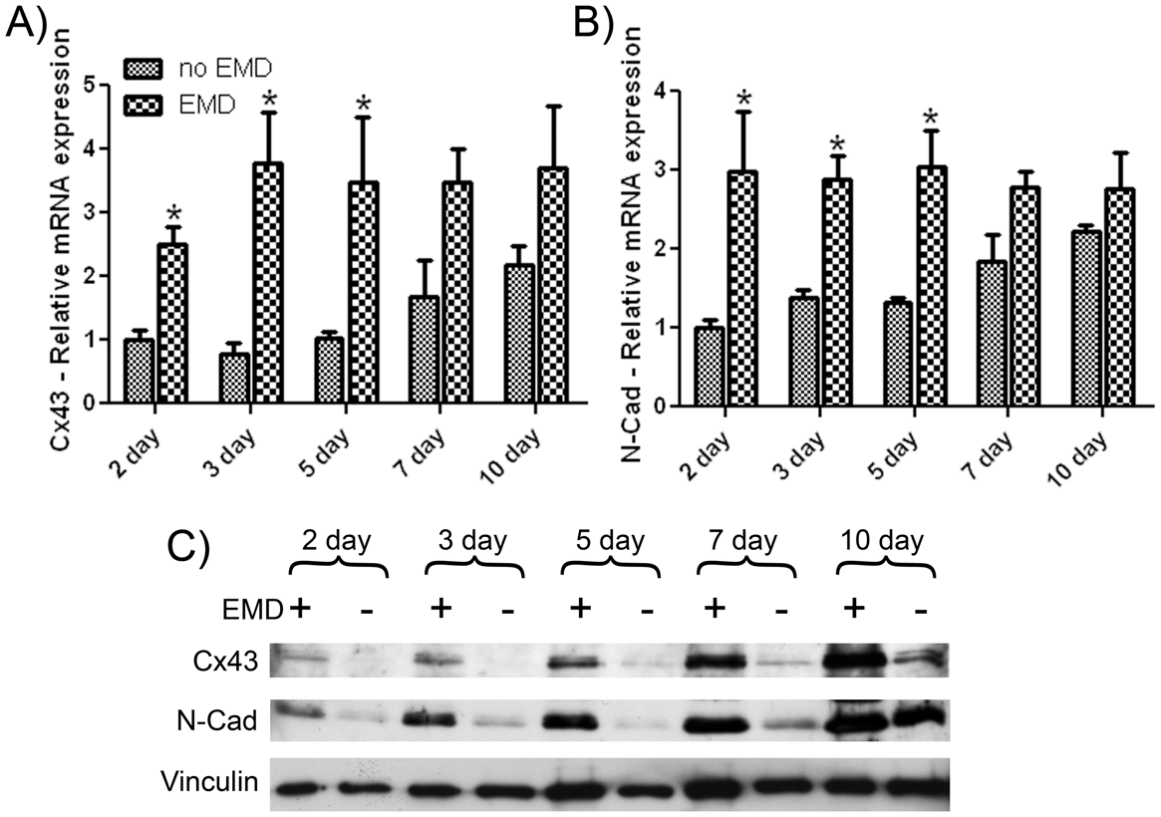
EMD increases osteoblast mRNA and protein levels of connexin 43 and N-cadherin. After 2, 3, 5, 7 and 10 days post seeding, mRNA was extracted and realtime PCR was performed using specific primers for connexin 43 (A) and N-cadherin (B). When samples were pre-coated with EMD, up to 4 fold increases in gene expression were observed for connexin 43 at 2, 3 and 5 days post seeding (A). 3 fold increases in gene expression of N-cadherin were also observed (B). Additional samples were extracted for western blot analysis (C). Elevated levels of both connexin 43 and N-cadherin were observed at 2, 3, 5 and 7 days post seeding. * denotes significant difference between EMD treated sample and respective control sample (p<0.05). Data shown is the average value from 3 independent experiments (3 replicates per experiment) ± SE.

Primary osteoblasts were also assessed for cx43 and N-cad gene expression at time points ranging from 2 to 10 days (Fig. 3). mRNA levels showed significant increases on EMD-coated surfaces at 2, 3 and 5 days post-seeding when compared to uncoated samples (Fig. 3A, 3B). At 7 and 10 days, higher (but no longer significantly different) expression of cx43 and N-cad was observed on EMD-coated samples when compared to control. Western blot analysis revealed similar patterns. At early time points following cell clustering, elevated expression of cx43 and N-cad were observed at 2, 3, 5 and 7 days post seeding on EMD-coated samples when compared to control (Fig. 3C). Differences in protein levels diminished as control samples reached cell confluence.

### Alkaline phosphatase activity

**Figure 4:**
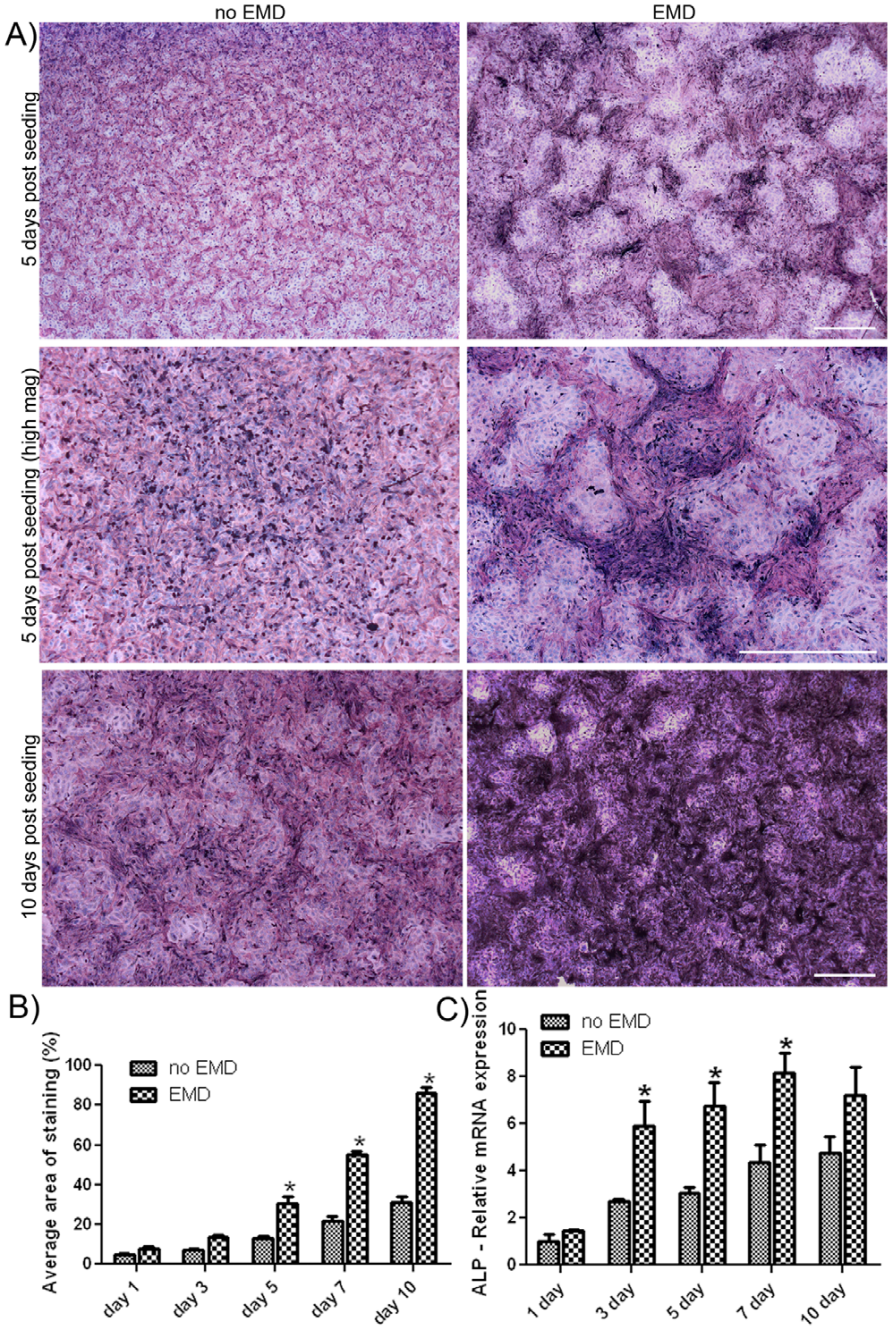
EMD increases alkaline phosphatase activity in osteoblast clusters. At time points 1, 3, 5, 7 and 10 days, osteoblasts were fixed and stained for alkaline phosphatase. A) EMD significantly increased alkaline phosphatase activity in clustered regions 5 days post seeding. After 10 days, complete staining was observed on samples coated with EMD when compared to control samples. (bar = 1000 µm) B) 10 fields of view per sample were captured and percentage area of staining calculated. Data represent means ± SE (results from 3 independent experiments). Significant increases in alkaline phosphatase activity were observed on EMD treated samples at 5, 7 and 10 days post seeding. C) mRNA was extracted and realtime PCR was performed using specific primers for alkaline phosphatase. Samples pre-coated with EMD showed significant mRNA levels 3, 5 and 7 days post seeding when compared to control groups (p<0.05). Data represent means ± SE (results from 3 independent experiments).

Osteoblasts seeded on EMD-coated samples showed significantly more alkaline phosphatase activity when compared to control samples (Fig. 4). 5 days post seeding, cells seeded on EMD surfaces displayed intense ALP staining (Fig. 4A). Interestingly, ALP staining was localized to cell clusters formed on EMD-coated samples. After 10 days, EMD coated samples showed complete ALP staining throughout their surfaces. Quantification of threshold staining showed significantly higher ALP activity on EMD-coated samples at 5, 7 and 10 days post seeding with elevated levels observed at all time points (Fig. 4B). mRNA expression of ALP activity was also significantly increased at 3, 5 and 7 days post seeding when compared to control samples (Fig. 4C).

### Quantification of osteoblast differentiation

**Figure 5:**
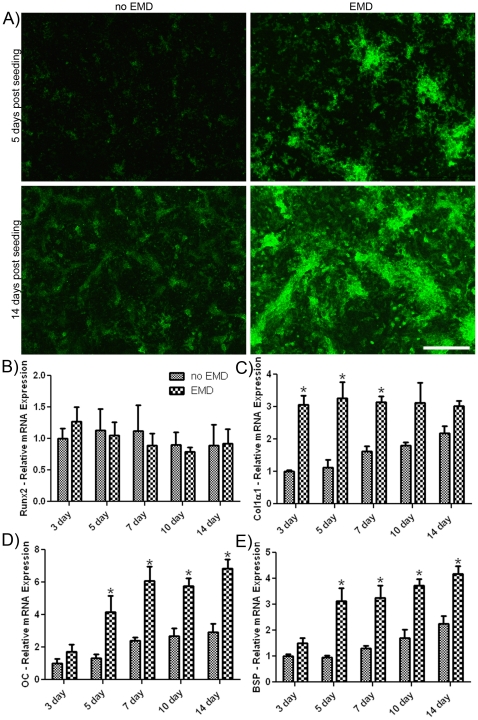
EMD increases extracellular matrix deposition of osteocalcin and osteoblast differentiation markers. At 5 and 14 days post seeding, human primary osteoblasts were labeled with specific antibodies to osteocalcin. Osteoblasts seeded on EMD-coated samples secreted higher levels of osteocalcin into the extracellular matrix when compared to control samples at 5 and 14 days (A) (bar = 200 µm). EMD-coated samples also increased mRNA levels of osteoblast differentiation markers (B–E). After 3, 5, 7, 10 and 14 days post seeding, mRNA was extracted and realtime PCR was performed using specific primers for Runx2, COL1A1, osteocalcin and bone sialoprotein. Levels of Runx2 were not significantly altered between EMD-coated and control samples (B). When samples were pre-coated with EMD, up to 3 fold increases in gene expression were observed for C) COL1A1, D) osteocalcin and E) bone sialoprotein (p<0.05). * denotes significant difference between EMD treated sample and respective control sample. Data shown is the average value from 3 independent experiments (3 replicates per experiment) ± SE.

Primary osteoblasts were assessed for OC immunostaining and gene expression of Runx2, COL1A1, OC and BSP at time points ranging from 3 to 14 days (Fig. 5). At 5 days, uncoated samples showed lower OC staining (Fig. 5A). When surfaces were precoated with EMD, osteocalcin staining was observed with extracellular deposition primarily within osteoblast clusters. At 14 days, control surfaces showed patterns of extracellular deposition that were more evenly dispersed across the entire surface. EMD coated surfaces displayed staining patterns localized to discrete nodules (Fig. 5A). Analysis of Runx2 gene expression showed no significant increase in mRNA levels at any time point, with or without EMD (Fig. 5B). By contrast, an initial significant increase in COL1A1 mRNA levels on EMD-coated surfaces was observed at 3, 5 and 7 days post seeding with elevated (but not significantly different) levels observed at 10 and 14 days (Fig. 5C). With respect to OC and BSP, up to a 3 fold increase in mRNA levels on EMD-coated surfaces at time points 5, 7, 10 and 14 days post seeding when compared to uncoated surfaces were observed (Fig. 5D, 5E). Interestingly, these increases in OC and BSP coincide with elevated expression of N-cad and Cx43 on EMD-coated samples.

### Von Kossa staining

**Figure 6:**
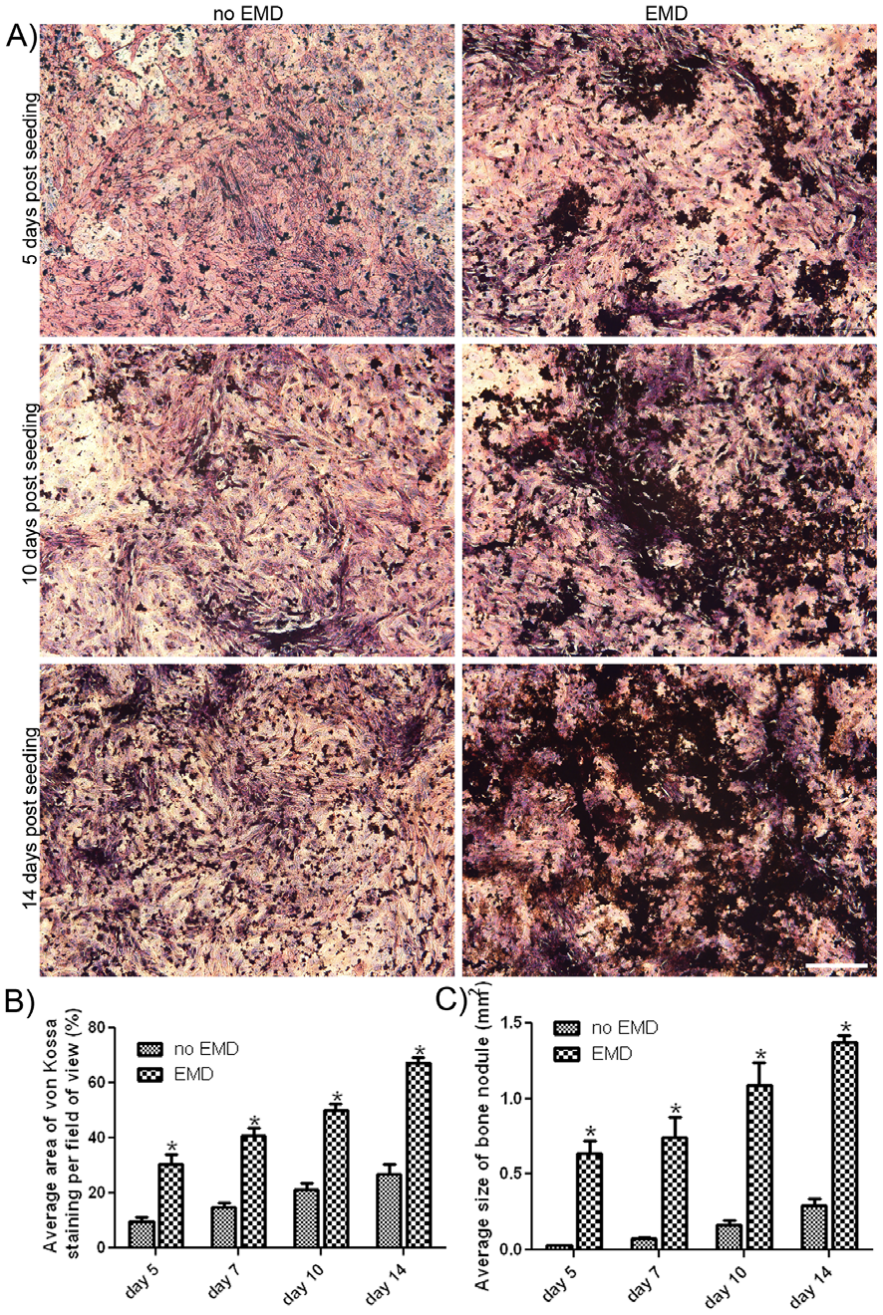
EMD significantly increases mineral deposition as assessed through von Kossa staining. At time points 5, 7, 10 and 14 days, primary human osteoblasts were fixed and stained with silver nitrate to determine patterns of mineralization. A) EMD significantly increased mineralization in clustered regions 5 days post seeding. At 10 and 14 days post seeding, areas of mineralization for EMD-coated samples were enlarged when compared to control samples (A) (bar = 1000 µm). 10 fields of view per sample were captured and percentage area of staining was quantified (B). At all time points, EMD significantly increased von Kossa staining. Furthermore, significant increases in nodule size were also observed at all time points (C). Data represent means ± SE (results from 3 independent experiments).

In order to determine whether early cell clustering may be indicative of early mineralized nodule formation, von Kossa staining was employed at time points 5, 7, 10 and 14 days (Fig. 6). Low levels of staining were observed on uncoated samples at both 5 and 10 days (Fig. 6A). On EMD-coated samples, the von Kossa staining surface area was increased especially in cell cluster regions (Fig. 6A). At 14 days post seeding, small nodules were formed on control surfaces whereas large coalescing clusters were observed on EMD-coated samples. Image analysis demonstrated significantly increased von Kossa staining (Fig. 6B) and nodule size (Fig. 6C) at all time points on EMD-coated samples.

## Discussion

In the past 15 years, a plethora of research has sought to advance our understanding of the functions of enamel matrix proteins (EMPs) have beyond amelogenesis. EMD has been previously shown to influence osteoblast differentiation using a wide range of cell models (MG-63, MC3T3-E1, Kusa/A 1, 2T9 cells, rat calvarial osteoblasts) [37], [40], [42], [43], [44]. EMD increased ALP activity [42], [45], mineral nodule formation [46] as well as markers for osteoblast differentiation such as BSP and OC [40], [43]. An unexpected observation in our previous study was the formation of cell clusters on EMD-coated surfaces after a period of 24 hours [40]. This phenomenon resembles mesenchymal condensation, which is a pivotal stage in the development of skeletal and other mesenchymal tissues. Condensation is the result of an active movement of previously dispersed cells that cluster together, differentiate and start to build tissues such as cartilage, bone, muscle, tendon, kidney and lung [7].

In this study, the formation of early cell clusters significantly increased cell-cell contact proteins cx43 and N-cad. In previous studies, the upregulation of N-cad mRNA levels has consistently been associated with early nodule formation and mineralization suggesting a role in bone formation [47], [48]. This phenomenon likely pertains to the signaling complexes associated with cadherins. Type 1 cadherins play an essential role in the structural organization and function of cells by linking cadherins through α- and β-catenin to the actin cytoskeleton [4], [49], [50]. N-cad also activates Wnt signaling which has been the focus of major investigation in recent years for osteoblast differentiation [5], [51].

An interesting observation in this study was the upregulation of the gap junction protein cx43 at similar time points as N-cad. The role of gap junctions in cell differentiation has also been extensively studied. In a variety of cell culture systems, cx43 induced osteoblast differentiation and mineralization [12], [14], [15], [52]. Interestingly, connexins retain an extremely short half-life of a few hours probably to respond to physiological requirements [21]. In this study, we found a quick increase in cx43 and N-cad expression as early as 24 hours post seeding on EMD-coated samples. Cx43 expression was maintained for up to 14 days. However, increases seemed to precede the expression of osteoblast differentiation markers such as ALP, OC and matrix mineralization. Gap junctional intercellular signaling could certainly explain these observations. Molecules transported through gap junctions, such as cAMP are known to play critical roles in the regulation of OC [53] and BSP gene expression [54] in osteoblasts. Furthermore, intracellular calcium fluctuations signal the activation of genes in different cell types [55]. There exist many examples on the dependence of cell coupling for the normal function of highly differentiated tissues is not exclusive to bone. Restoration of gap junctional communication in thyroid cells increases thyroglobulin expression [56]. Steroidogenic response of bovine and human adrenal fasciculo-reticularis cells to corticotrophin was dependent on junctional intercellular communication [57] and muscle cell contraction in response to alpha1-adrenergic stimulation was dependent on cell coupling [58]. Results from the present study further support the view that gap junctional communication is important for cell differentiation.

The ability for osteoblasts to respond to biomaterials such as EMD shows further promise for tissue engineering applications in periodontal and alveolar defects. An interesting aspect which remains unsolved is the ability for EMD to promote cell cluster formation. The possibility exists that EMD may contain chemokinetic proteins or may stimulate cells to produce chemotactic factors that attract other cells. In this regard, EMD is thought to contain bone morphogenetic proteins (BMPs) [59], [60] and transforming growth factor β [61], [62]. BMPs have been the focus of intense investigation for clinical application and studies have demonstrated a role for BMPs in the formation of many organs and patterning of the skeleton by inducing the formation of mesenchymal condensations [63], [64].

In the present study, it was observed that Runx2 mRNA levels remained unchanged. Other authors have also investigated the influence of EMD on gene expression of Runx2 and have not observed any significant differences [40], [42], [43], [45]. It has previously been reported by Komori et al. that Runx2 is necessary for early osteoblast differentiation however at later time points, inhibits immature osteoblasts from differentiating into mature osteoblasts and osteocytes [65]. Given that the primary cells isolated in the present study were isolated from alveolar bone, it is likely that these cells were comprised primary of pre-osteoblasts and/or osteoblasts which had already differentiated past the mesenchymal stem cell phenotype and no longer require Runx2 gene expression.

More recent research has focused on the roles of different proteins found in EMD. Mumulidu et al. used high performance liquid chromatography to fractionate EMD into three major components: a 20 kDa, 12+9 kDa and 5 kDa fractions [66]. Amelogenin (20 kDa), the major component in EMD is an adhesion molecule that was initially thought to be responsible for the effects observed in EMD [67]. Further analysis has revealed that the 5 kDa component of EMD also contains many bioactive molecules [66]. More recently, Johnson et al. observed that each of the 3 fractions stimulates different cellular mechanisms [68]. These findings suggested roles for multiple proteins in the attachment, proliferation and differentiation of osteoblasts by EMD.

To clarify the roles of individual proteins on cellular mechanisms, the use of recombinant EMPs have been studied. Amelogenin, the main component of EMD, increases cell adhesion [67], proliferation [69] and differentiation [70], [71]. It also binds to heparan sulfate and BMP2 [72] and uses the β-catenin and Wnt pathways [73]. Recent research with recombinant ameloblastin, a second protein found in EMD, concluded that not only amelogenin has growth factor-like activity [74], [75]. Still, the combination of proteins and growth factors present in EMD seems to improve the *in vitro* outcomes over individual recombinant proteins.

In conclusion, the observations from this study indicate that upregulation of cell-cell contact proteins at early stages in osteoblasts seeded on EMD-coated samples might be a mechanism for accelerating bone formation. Gap junctional intercellular communication constitutes a fundamental mechanism of differentiated cells and communication through channels which directs the control and regulation of gene expression and provides a means to respond cooperatively to a stimulus. Furthermore, the clinical evidence of EMD supports the hypothesis that gap junctional communication provides a means for a group of fully differentiated cells to perform a function coordinately not only in bone, but also in other tissues.
